# Pandemic management impacts Slovak health care workers’ quality of life during the second wave of the COVID-19 pandemic

**DOI:** 10.1371/journal.pone.0283740

**Published:** 2023-03-30

**Authors:** Veronika Pacutova, Andrea Madarasova Geckova, Peter Kizek, Sara Maria Majernikova, Andrea F. de Winter, Sijmen A. Reijneveld

**Affiliations:** 1 Faculty of Medicine, Department of Health Psychology and Research Methodology, P. J. Safarik University, Kosice, Slovakia; 2 Department of Community & Occupational Medicine, University Medical Center Groningen, University of Groningen, Groningen, Netherlands; 3 Faculty of Social and Economic Sciences, Institute of Applied Psychology, Comenius University Bratislava, Bratislava, Slovakia; 4 Faculty of Medicine, Clinic of Stomatology and Maxillofacial Surgery, University Hospital of Louis Pasteur, P. J. Safarik University, Kosice, Slovakia; 5 Faculty of Life Sciences, Division of Biosciences (student), University College London, London, United Kingdom; George Mason University, UNITED STATES

## Abstract

**Background:**

The COVID-19 pandemic led to accepting a lot of various protective pandemic management-related measures (PanMan), which may have had a large impact on health care workers (HCWs) but evidence is scarce. We therefore explored the impact of measures during the second wave. We assessed the associations of PanMan with the Quality of Life (QoL) of hospital HCWs.

**Methods:**

We collected data from 215 HCWs (77.7% females, mean age 44.4), who were working at the COVID-related departments of one large hospital in eastern Slovakia via a questionnaire, specifically developed in direct collaboration with them. We assessed PanMan related factors, such as COVID-19 experience, information overload, non-adherence of the public, work stress, barriers and facilitators of health care provision, and QoL related factors, such as impact on family life and activities, housekeeping, relationships with relatives and mental well-being. To analyse the data, we used logistic regression models adjusted for age and gender.

**Results:**

PanMan greatly impacted the QoL of HCWs, in particular family life, housekeeping and mental well-being (odds ratio, 6.8–2.2). The most influential PanMan factors were COVID-19 experience (3.6–2.3), work stress (4.1–2.4) and barriers in health care provision (6.8–2.2). Perceiving work stress had a negative impact on all QoL domains, even on relationships with the greatest impact. Conversely, the PanMan factors reducing the negative impact on QoL were training and colleagues’ support (0.4–0.1).

**Conclusion:**

PanMan had a strong negative impact on the QoL of hospital HCWs during the second wave of the COVID-19 pandemic.

## Introduction

The second wave of the COVID-19 pandemic (further: the pandemic) led to a significant increase in infected and hospitalised patients, which seriously impacted worldwide institutional health care. Hospital health care workers (HCWs) working directly with COVID-19 patients are among the frontline workers who perceived the high risks of the shared work environment [1,2]. They may perceive the pandemic as a new, traumatic stressor, especially due to experiencing exceptional stress, workloads, grief, stigmatization, high mortality rates and risk of infection [3,4]. As we already know, their risk of getting infected is 7-times higher than non-HCWs, but in contrast is associated with lower hospitalisation and mortality [5,6]. Women and nurses are at the highest risk of getting infected, while men and doctors are at the highest risk of fatality [7]. In Slovakia, they experienced much higher risks compared to the first wave, because of increased numbers of COVID-19 patients (i.e. 2690 registered in the first wave vs. 359 874 in the second wave) [8]. In general, the incidence and mortality, and the severity of COVID-management measures as measured by the Oxford stringency index during the second wave were higher in Slovakia compared to other European countries, which may be due to a lower vaccination rate [9].

To contain this rapidly spreading disease, global politicians approved protective pandemic management-related measures (PanMan), which were intended to stop the virus and to protect HCWs. However, they accounted relatively little for the possible impact of the pandemic or pandemic-related measures on HCWs’ quality of professional and personal life (QoL). The QoL of HCWs decreased during the pandemic, especially compared to the general population [10]. PanMan supported the pre-existing stressful work environment by work pressure and workload, which may have seriously contribute to them decreasing. On one hand, their QoL may have been affected by measures, such as home isolation, quarantine, the closing of educational institutions, non-essential stores, the checking of body temperatures, lockdowns, the necessity to wear face masks and the use disinfection. On the other hand, the quality of their professional life could also have been influenced by key challenges, such as the use of personal protective equipment, a lack of hospital beds, a lack of staff, work exhaustion and the need of to redesign methods of care [11–14]. We previously analysed the impact of the first wave of PanMan on the QoL of ambulatory HCWs, i.e. dentists. We found that PanMan greatly impacted, e.g. their financial situation due to reduced treatments, mental health due to intensive unknown stressful situations, and housekeeping due to different restrictions in household management, shopping, cooking and childcare [15]. Based on that, we decided to collect another round of data reflecting the second wave of the pandemic, but for that purpose we approached hospital HCWs treating COVID-19 patients directly and facing the burden of the second wave.

Generally, little is known about how PanMan can negatively impact the QoL of HCWs and alternatively what is helping to reduce it. Regarding the results from the first wave, we expected that PanMan would also negatively impact the QoL of hospital HCWs in the second wave of the pandemic. Therefore, we aimed to assess the associations of PanMan with the QoL of hospital HCWs at COVID-related departments during the second wave of the pandemic.

## Methods

### Sample and procedures

We invited all practicing HCWs from the COVID-related departments (e.g. infection/anaesthesiology and intensive care/pathology) of one hospital (covering the Kosice region), one rescue service (covering the Kosice region) and one dialysis service (covering the whole of Slovakia) to participate in a cross-sectional study during the second wave of the pandemic through their employing institution. Data were collected via an online or paper-based version of questionnaire from May to September 2021. In total, we received 233 responses, which covered around 8% of the overall number of employees addressed. We could not compute a response rate, because we did not approach the HCWs personally, but the invitation was distributed through their employing institution (bulletin boards, web, announcements for employees). Subsequently, we excluded those who did not report their gender (n = 6) and who did not specify their profession (n = 12). Our final sample consisted of 215 respondents (77.7% females, mean age/SD = 44.4/±10.2). We developed a questionnaire specifically in direct cooperation with interviewed representatives of the participating hospital HCWs and based on a literature review. We arranged appropriate measurements to cover it, considering their opinions, and the final version was piloted in a small sample of HCWs to assure clarity and suitability. In general, the questionnaire covered eleven different areas: sociodemographic information, exposure to COVID-19, risk perception/acceptance/stigma/vulnerability, information overload, non-adherence to pandemic measures, impact on health care provision, barriers and facilitators of health care provision, impact on QoL, adverse events, help and support provided, personal coping resources. This questionnaire is shown in the Appendix A in A1 File.

### Ethics

The study was approved by the Ethics Committee of the Faculty of Medicine at P. J. Safarik University (14N/2020) and the Ethics Committee of Health care Providers (2021/EK/05031; 813/2021). All data and information gathered from the documentation, including demographic and clinical data, were used in accordance with the ethical standards as laid down in the 1964 Declaration of Helsinki and its later amendments or comparable ethical standards. Written informed consent to participate in this study was provided by all participants.

### Measures

#### Pandemic management-related measures (PanMan)

PanMan covered several issues related to COVID-19 and public management of the pandemic. COVID-19 experience was measured by asking HCWs if they had experience with serious COVID-19 occurrence accompanied by hospitalisation or death personally, among close relatives, or within their work team. Information overload was measured as how much HCWs were concerned about pandemic news and how frequently they followed it during the second wave of the pandemic from January till March 2021. Similarly, we measured the non-adherence of the public, which consisted of how frequently they saw other people not following pandemic measures during this period and how much they were concerned about it. Work stress measured if HCWs were concerned due to providing triage, applying work orders, limitations due to emergency status or performing their work without specialisation from January till March 2021, and we further analysed those who reported at least one of the designated work stressors. Barriers and facilitators of health care provision regarding the factors hindering or helping HCWs in providing health care during the second wave of pandemic from January till March 2021. Barriers regarded (a) use of personal protective equipment (PPE), (b) lack of hospital beds, (c) lack of staff, and (d) work exhaustion. Answers were dichotomised as partially/not limited vs. totally/significantly limited for each subcategory. Facilitators regarded (a) efficient department management, (b) colleagues’ support, (c) training, and (d) public solidarity manifestation. Answers were dichotomised as highly vs. slightly/a little/not at all helpful (**Appendix A in S1 File**).

#### Quality of Life (QoL)

QoL was measured by asking HCWs: Did difficulties in providing health care due to introducing pandemic management affect your (a) family life and activities, (b) housekeeping, (c) relationships with relatives, (d) financial situation and (e) mental well-being during the second wave of pandemic? Answers on a Likert scale were dichotomised into those reporting significant worsening vs. those reporting slight worsening/significant/slight improving and no change of a particular area of life (**Appendix A in S1 File**).

### Statistical analysis

First, we used descriptive statistics to characterise the background of our sample. Second, we described PanMan (COVID-19 experience, information overload, non-adherence of the public, work stress, barriers and facilitators of health care provision) and QoL (family life and activities, housekeeping, relationships with relatives and mental well-being) of hospital HCWs via prevalence data. Third, we used logistic regression models, adjusted for age (as centred continuous level variable, age and age squared) and gender, to assess the associations of PanMan factors with QoL (financial situation was excluded because of insufficient numbers of HCWs responding) [16]. As a result, we reported an odds ratio (OR) with the 95% confidence interval (CI) for each outcome and used IBM SPSS Statistics 23 for Windows.

## Results

### Background characteristics

The majority of HCWs in our sample were females, 77.7% (n = 167), and more than a half regarded as nurses 52.1% (n = 112); and 32.1% (n = 69) regarded as doctors. For more details, see **Table 1**.

**Table 1:** Demographic characteristics of the respondents (Slovakia 2021; n = 215 HCWs).

Variables	N (%)
**Age (mean/SD)**	44.4/±10.2
**Gender**	
Women	167 (77.7)
Men	48 (22.3)
**Profession**	
Nurses	112 (52.1)
Doctors	69 (32.1)
Rescuers	27 (12.6)
Other HCWs	7 (3.3)
**Department of HCWs**	
*Hospital–local*	
Infection department	46 (21.4)
Anaesthesiology and Intensive Care department	25 (11.6)
Pathology department	22 (10.2)
*Hospital–local and serving other hospitals*	
Dialysis department	92 (42.8)
Rescue department	30 (14.0)

### Exposure to PanMan and impact on QoL

HCWs reported work exhaustion (47%), lack of staff (35%), use of PPE (34%) and lack of hospital beds (31%) as the most significant barriers in health care provision. In contrast, they reported efficient colleagues’ support (60%) and department management (50%) as the most facilitating PanMan factors helping them to handle the pandemic. A total of 40% of them perceived at least one work stressor. Limitations due to emergency status concerned them the most (70%), followed by applying work order (56%), providing triage (37%) and working without appropriate specialisation (12%). A majority reported a significant worsening of QoL in mental well-being, family life and activities, and housekeeping, while 50% also had a serious COVID-19 experience. For more details, see **Table 2**.

**Table 2:** Description of PanMan factors and QoL of Slovak hospital HCWs during the second wave of the pandemic.

Variables	N (%)
**Pandemic management (PanMan)**	
***COVID-19 experience***	
Had a serious COVID-19 experience (due to hospitalisation or death)	108 (50.2)
***Information overload***	
Did not follow **and** not concerned	127 (59.3)
Followed the news **or** highly concerned	65 (30.4)
Followed the news **and** highly concerned	22 (10.3)
***Non-adherence of the public***	
Never or sometimes saw non-adherence **and** did not concerned	81 (37.9)
Almost always/always saw non-adherence **or** highly concerned	75 (35.0)
Almost always/always saw non-adherence **and** highly concerned	58 (27.1)
***Work stress***	
At least 1 stressor (triage, work order, emergency status, no specialisation)	84 (40.0)
***Barriers of health care provision***	
Use PPE^1^	72 (33.5)
Lack of hospital beds^1^	65 (30.7)
Lack of staff^1^	75 (35.0)
Work exhaustion^1^	101 (47.4)
***Facilitators of health care provision***	
Efficient department management^2^	107 (50.0)
Colleagues’ support^2^	129 (60.3)
Training^2^	64 (30.2)
Public solidarity manifestation^2^	50 (26.2)
**Quality of Life (QoL)**	
Significant worsening of Family life and activities	43 (20.1)
Significant worsening of Housekeeping	29 (13.6)
Significant worsening of Relationships with relatives	12 (5.6)
Significant worsening of Financial situation*	3 (1.4)
Significant worsening of Mental well-being	54 (25.2)

^1^ totally/significantly limited

^2^ highly helpful

*excluded from further analyses.

### Associations of PanMan with QoL

Associations of PanMan with QoL were strongest regarding family life and activities, housekeeping, and mental well-being, while conversely weakest for the HCWs’ relationships with relatives. The PanMan factors with the strongest associations regarded COVID-19 experience (odds ratio, OR, varying between 3.6–2.3), work stress (OR varying between 4.1–2.4) and barriers of health care provision (OR varying between 6.8–2.2). Following and being concerned about pandemic information had a negative impact only on their family life and activities QoL, as did non-adherence of the public. The latter also had impact on their mental well-being QoL. Perceiving at least one stressor was associated with all domains of QoL, and greatly impacted their relationships (OR 4.1). On the one hand, we found that barriers in health care provision, such as use of PPE, lack of hospital beds, lack of staff and work exhaustion, negatively affected QoL. On the other hand, we found that facilitators, such as colleagues’ support and training, were able to significantly reduce this negative impact (OR varying between 0.4–0.1), but we did not confirm a positive impact of efficient department management and public solidarity manifestation. For more details, see **Table 3**.

**Table 3:** Associations of PanMan with QoL of hospital HCWs during the second wave of the pandemic, logistic regression analyses adjusted for age and gender with odds ratios (OR) and 95% confidence interval (95%CI).

PANDEMIC MANAGEMENT (PanMan)	QUALITY OF LIFE (QoL)			
	Family Life and activities	Housekeeping	Relationships with relatives	Mental well-being
**COVID-19 experience**				
Serious experience	**2.8(1.32–5.74)** ^**^	**3.6(1.43–8.87)** ^**^	0.9(0.29–3.03)^ns^	**2.3(1.21–4.50)** ^*^
**Information overload**				
Followed or concerned Followed and concerned	2.0(0.92–4.21)^ns^ 2.9(1.03–8.24)^*^	1.6(0.69–3.74)^ns^ 0.8(0.17–4.07)^ns^	3.1(0.82–11.5)^ns^ 3.2(0.55–18.9)^ns^	1.5(0.77–3.09)^ns^ 1.9(0.67–5.11)^ns^
**Non-adherence of the public**				
Non-adherence or concerned Non-adherence and concerned	2.0(0.85–4.75)^ns^ 2.6(1.05–6.27)^*^	1.7(0.66–4.61)^ns^ 1.9(0.66–5.30)^ns^	2.2(0.40–12.6)^ns^ 4.8(0.92–24.6)^ns^	**2.8(1.21–6.24)** ^*^ **4.0(1.71–9.36)** ^***^
**Work stress**				
At least one stressor	**2.4(1.20–4.87)** ^*^	**2.8(1.20–6.50)** ^*^	**4.1(1.04–15.9)** ^*^	**3.6(1.83–6.90)** ^***^
**Barriers of health care provision**				
Use of PPE	**2.8(1.39–5.60)** ^**^	**3.5(1.53–7.91)** ^**^	2.1(0.65–6.75)^ns^	1.7(0.88–3.20)^ns^
Lack of hospital beds	**3.7(1.82–7.53)** ^***^	**5.2(2.21–12.0)** ^***^	2.4(0.73–7.68)^ns^	**2.6(1.36–5.09)** ^**^
Lack of staff	1.9(0.97–3.88)^ns^	**2.6(1.14–5.81)** ^*^	0.9(0.26–3.04)^ns^	**2.2(1.18–4.26)** ^*^
Work exhaustion	**2.9(1.41–6.04)** ^**^	**6.8(2.45–18.9)** ^***^	3.5(0.91–13.2)^ns^	**4.4(2.19–8.89)** ^***^
**Facilitators of health care provision**				
Efficient department management	0.8(0.39–1.60)^ns^	0.5(0.23–1.22)^ns^	0.7(0.19–2.16)^ns^	1.0(0.52–1.85)^ns^
Colleagues’ support	**0.4(0.20**–0.82)^*^	0.5(0.23–1.18)^ns^	0.7(0.20–2.14)^ns^	0.7(0.38–1.35)^ns^
Training	0.5(0.22–1.17)^ns^	**0.1(0.03–0.61)** ^**^	0.8(0.20–2.93)^ns^	**0.4(0.20–0.93)** ^*^
Public solidarity manifestation	0.9(0.37–2.01)^ns^	0.4(0.13–1.20)^ns^	1.4(0.40–4.90)^ns^	0.7(0.32–1.55)^ns^

Note

*** p≤0.001

** p<0.01

* p<0.05; ^ns^ non-significant.

## Discussion

The second wave of the pandemic and its management had a large impact on hospital HCWs’ QoL, in particular on family life and activities, housekeeping and mental well-being. Other studies also reported a negative impact of the pandemic outbreak on physical, mental and social well-being (QoL) of HCWs using various measurement instruments, for instance showing increased risks of burnout [13,17–23]. Our study showed that the negative impact of the pandemic on mental well-being QoL may be explained by increased work stress (fear, workload, pressure, exhaustion), which may cause psychological sequelae (anxiety, depression, depersonalisation, posttraumatic stress syndrome or disorder) and sleep difficulties [24–35]. Poor mental well-being and a high level of exhaustion could even lead to burnout or job leaving intentions [27,34,36–40]. There are also studies showing that their poor mental well-being even before the pandemic strongly co-influenced other domains of QoL, and this could even have been exacerbated by the pandemic situation [14,37,38]. The underlying mechanism may regard family imbalance due to the increased need to care and worry about children or for the elderly, family worries regarding the own health condition, home-schooling, social isolation, stigmatisation and limited leisure activities outside [13,24,28,40,41], thus affecting family life. Similarly, difficulties with food supplies, shopping, cooking and maintaining physical distance [28,41] may have decrease housekeeping QoL as well. We learned that PanMan had a significant impact on the personal and professional life of HCWs, with the essential impact of work stress on mental well-being, which may also have strongly co-influenced other domains of QoL and led to burnout or job leaving intentions.

We found that an especially serious COVID-19 experience, work stress and barriers in health care provision affected the QoL of hospital HCWs. Regarding personal COVID-19 experience, a mechanism may be that our HCWs felt lonely, angry, guilty and worried about their own health and about the high possibility of spreading the infection, even to their relatives [24,28,29,35,41–43]. In a study by Nohesara et al., HCWs expressed feelings of guilt for being possible carriers, due to lack of treatment facilities and vaccinations, and for not having the chance to take care of the loved one [44]. Also, feelings of public stigmatisation could have played a significant role, as could have financial concerns, depending on who the family breadwinner was [13,41]. The high mortality due to coronavirus could have deepened any worries, especially for those working in direct contact with infected patients [18,44]. Regarding COVID-19 experience of their closest or co-workers, they might have experienced their own suffering, increased care provision, mandatory home-isolation following close contact, or no possibility to visit or say a final goodbye to them in fatal cases [13,24]. Additionally, in the case of co-workers’ experience, this could also have had a serious impact due to understaffing or increased exhaustion, which may also have been considered as barriers to health care provision [13,36,41]. Regarding work stress, selection and prioritising patients to provide care could have been very stressful due to the moral and legal consequences of providing the greatest good for the greatest number or belated guidelines on how to do it [26,45]. Moreover, HCWs had no possibility to take time off, vacation, resign or refuse redeployment [26,31,36]. Regarding health care provision, the use of PPE could have caused dehydration, overheating, discomfort, pain or worse communication [21,25,32]. They reported consternation about frequent changes in PPE, not having proper breaks (“wobble” rooms) and even struggling with a lack of hospital beds for patients. All of these limitations may have contributed to the negative impact of significant worsening their QoL. We learned that hospital HCWs working at COVID-related departments may have struggled even more than other HCWs, which should have resulted in higher support of their resilience.

Perceiving at least one stressor in the work environment had a negative impact on all domains of QoL, with the greatest impact on relationships. HCWs usually encounter stressors from various sources within their work [10,13,14,25,30–32,36,37,41]. First, they might perceive essential work stress because of the origin of their work, such as long hours and night shifts, a heavy workload (responsibilities, patients, pressure loads), dealing with pain and emotional distress, and facing a shortage of HCWs [14,36,37]. Second, they may have experienced additional PanMan stress due to new pandemic occupational changes, such as redeployment (loss of team and routine), no possibility of taking proper breaks or time off, a lack of preparedness, non-efficient management, poor working conditions (safety concerns, lack of and use of PPE, disinformation, ambiguity of performance), lack of effective treatments specifically for COVID-19, and death of their patients [10,13,25,30–32,40,41,43]. Evidently, experiencing these various stressful work and PanMan circumstances may have accumulated and thus could have had an impact on all domains of QoL. As a result of our study, we found that perceiving work stress also had an impact on relationships with relatives (QoL). Another study, by Pieh et al., showed that quality of relationships is related to QoL, not relationship per se [47]. This may mean that those who have poor quality relationships might perceive a higher level of work stress. We learned that PanMan may have escalated work stress, which might have even accumulated, thus testing the quality of HCWs’ relationships.

We further found that facilitators, such as colleagues’ support and training, seemed to reduce the negative impact of PanMan on QoL, in particular family life, housekeeping and mental well-being. Generally it is known, that if HCWs experience more social support from friends they have a higher QoL, especially regarding physical health [10]. HCWs further reported that peer support and a “buddy system” helped more than professional psychological support, which was usually offered during their shifts, meaning that they could not attend [45,46]. They pointed out that maintaining the consistency of working teams, which may facilitate colleagues helping one another, was important. Moreover, training, gaining new experience and good management communication diminished their concerns and worries [47]. We learned that we should promote “peer support” and strengthen the unification of colleagues’ relations in order to decrease HCWs’ burden and offer them the training for professional and personal growth.

### Strengths and limitations

The main strength of this study is that we had the possibility to reach a representative sample of HCWs from COVID-related departments during the second wave of the pandemic. Thanks to that we were able to gather information about PanMan, such as COVID-19 experience, information overload, non-adherence of the public, work stress, barriers and facilitators of health care provision, and about QoL, such as impact on family life and activities, housekeeping, relationships with relatives and mental well-being.

Furthermore, some limitations need to be considered. First, our sample was relatively small and came from only one hospital. By focusing intensively on one hospital, we were able to cover a full variety of departments and specialists involved in the pandemic. Nevertheless, those experiencing a higher burden might participate less in research, so our findings probably regard an underestimation of the real effects. Second, our study used a cross-sectional design, so no causal relations can be established between PanMan and QoL. Third, we mostly used self-prepared questions, which adds to the study’s acceptance but also implies that information bias may have occurred.

### Implications

Our finding that PanMan had a serious impact on the QoL of HCWs implies that there is a need to raise awareness of this negative impact to stimulate adequate and effective actions and policies to prevent negative outcomes. Potential strategies could be to promote resilience, mitigate identified barriers, improve cognitive reappraisal and support the strengthening of relationships among colleagues [48]. The study by Halms et al. made recommendations for mental health support of HCWs, such as “help hotlines”, “self-care”, “identification/monitoring of individuals at higher risk” and “access to mental health services”. These apply to our findings as well [46]. HCWs should be enabled to make use of mental health services, and the professionals working in these services need to be involved in developing guidelines to relieve their burden [49]. Also help from psychiatrists, e.g. in developing self-help, group or individual support or from paraprofessionals to relieve distress, e.g. via brief psychological interventions should become available for them [50,51]. Regarding HCWs’ work exhaustion and work stress, it might help to create a safe, employee-oriented work environment (by providing enough PPE, trainings for supervisors, specialized trainings for HCWs and by promoting professional development), ensure the safety of clinical procedures, cancel non-essential procedures to save the workforce, provide more breaks per day, provide a “wobble” room, change shifts weekly at COVID-related departments, allow HCWs to take their time off, use pandemic adjusted staffing and even find new part-time workers for basic duties [46,52–54]. Above all, it is strongly recommended that the QoL of HCWs and their families be routinely monitored on an institutional level to prevent burnout and minimise their job leaving intentions in general [55].

## Conclusions

PanMan had a strong negative impact on the QoL of hospital HCWs during the second wave of the pandemic, in particular due to COVID-19 experience, work stress and barriers in health care provision, and facilitators, such as colleagues’ support and training, might mitigate it.

## Supporting information

S1 File Appendix A. Questionnaire used to collect data among Slovak health care workers during the second wave.
(DOCX)
