# Supplementary material for: Pandemic management impacts Slovak health care workers’ quality of life during the second wave of the COVID-19 pandemic
Source: PLoS One. 2023 Mar 30;18(3):e0283740. doi: 10.1371/journal.pone.0283740 (PMC10062551; doi:10.1371/journal.pone.0283740)
Supplement: S1 File — Questionnaire used to collect data among Slovak health care workers during the second wave. (DOCX) [file pone.0283740.s001.docx]

# Appendix A

**Pandemic Management (PanMan):**

**COVID-19 experience**

Q.1 Did you have any serious experience of COVID-19 disease accompanied by hospitalisation or death?

- Me/my family/my co-workers had a serious COVID-19 experience due to hospitalisation or death
- Me/my family/my co-workers had a COVID-19 experience but without hospitalisation or death.
- I did not have such an experience

**Information overload**

Q.1 Did you follow the pandemic news during the second wave (from January till March 2021)?

- Yes, several times per day
- Yes, at least once a day
- Yes, but not more than usual
- No

Q.2 Were you concerned about the pandemic news?

- No
- A little concerned
- Highly concerned

**Non-adherence of the public**

Q.1 How often did you meet a patient or another person who did not follow the pandemic measures (e.g. did not wear face masks) from January till March 2021?

- Never
- Sometimes
- Almost always
- Always

Q.2 Were you concerned about the non-adherence of the public (not wearing face masks)?

- No
- A little concerned
- Highly concerned

**Work stress**

Q.1 From January till March 2021, were you ever concerned about:

(a, yes; b, no)

- Providing patient triage
- Applying a work order
- Limitations due to emergency status (no possibility to take vacations or resign)
- Performing work duties without specialisation

**Barriers of health care provision:**

Q.1 How much did the following circumstances hinder you in providing health care (from January till March 2021)?

(a, totally limited; b, significantly limited; c, partially limited; d, not limited)

- Use of PPE
- Lack of staff
- Lack of hospital beds
- Work exhaustion in the team

**Facilitators of health care provision:**

Q.1 How much did the following circumstances help you in providing health care (from January till March 2021)?

(a, highly; b, slightly; c, a little; d, not at all)

- Efficient department management
- Colleagues’ support
- Training
- Public solidarity manifestation

**Quality of Life (QoL):**

Q.1 Did difficulties in providing health care due to introducing the pandemic management affect:

(a, significantly improved; b, slightly improved; c, did not change; d, slightly worsen; e, significantly worsen)

- Your family life and activities
- Your housekeeping
- Your relationships with relatives
- Your financial situation
- Your mental well-being
